# Electrical Properties of Ultra-Fast 3D-Trench Electrode Silicon Detector

**DOI:** 10.3390/mi11070674

**Published:** 2020-07-10

**Authors:** Manwen Liu, Tao Zhou, Zheng Li

**Affiliations:** 1 Institute of Microelectronics, Chinese Academy of Sciences, Beijing 100029, China; 2 School of Materials Science and Engineering, Xiangtan University, Changsha 411109, China; zt15773241305@163.com; 3 College of Physics and Optoelectronic Engineering, Ludong University, Yantai 264025, China; 4 School for Optoelectronic Engineering, Zaozhuang University, Zaozhuang 277160, China

**Keywords:** ultra-fast 3D-trench electrode detector, electric filed, hole concentration distribution, full depletion voltage, induced current, drift time, high position resolution

## Abstract

In our previous work on ultra-fast silicon detectors, extremely small carrier drift times of 50–100 picoseconds were predicted for electrode spacing of 5–10 μm. Expanding on these previous works, we systematically study the electrical characteristics of the ultra-fast, 3D-trench electrode silicon detector cell with p-type bulk silicon, such as electric potential distribution, electric field distribution, hole concentration distribution, and leakage current to analyze the full detector depletion voltage and other detector properties. To verify the prediction of ultra-fast response times, we simulate the instant induced current curves before and after irradiation with different minimum ionizing particle (MIP) hitting positions. High position resolution pixel detectors can be fabricated by constructing an array of these extremely small detector cells.

## 1. Introduction

The applications of high energy and/or position resolution silicon detectors are very broad, including medical imaging [1,2], aerospace work [3,4,5], high energy physics experiments [6,7,8], and nuclear safety guards, as well as in many fields with scientific applications such as photonics and astrophysics [9,10,11]. With the upgrade of the Large Hadron Collider (LHC) in the European Organization for Nuclear Research (CERN) to the High-Luminosity Large Hadron Collider (HL-LHC), the radiation fluence in the structure has already increased to 1 × 10^16^ n_eq_/cm^2^ (1 MeV neutron equivalent per square centimeter) [12,13]. Scientists have to find new ways to improve detectors’ radiation hardness within this environment by either finding new materials [14,15] or designing new detector structures [16,17]. Silicon detectors (including stripe detectors, pixel detectors, silicon drift detectors, and 3D electrode detectors) have many advantages, such as high energy resolution, quick response time, and ease of very large scale integration (VLSI), etc. [18,19,20,21]. Silicon detector technology has also improved and is now able to fabricate 3D detectors [22]. The 3D-trench detector overcomes the disadvantages of the traditional 3D-column electrode detector, such as the electric potential saddle point, a low electrode field area in the geometric center of the column electrode [23,24]. To pursue high radiation hardness and ultra-fast response time, as well as high energy and position resolution, we propose an ultra-fast silicon detector based on the 3D-trench electrode detector [25,26].

With the proposal of the ultra-fast silicon detector, picosecond-level response time can be attained. By numerical calculations and full 3D simulations of electric characteristics, such as electric potential and field, we aimed to investigate the minimum bias voltages needed for carriers, generated by incident particles and photons, to reach the saturation drift velocity in the most parts of the detector volume. Often, the carriers reach saturation velocity in the most parts of the detector under only a few volts of bias voltage, so we predicted that a carrier drift time of about 50 ps can be reached for a detector with an electrode spacing of 5 μm [25,26]. During the course of this work, we simulated a 3D funnel shape of the electric potential distribution to determine if electrons funnel into the collection electrode to be collected, even in a radiation environment. However, in our previous work, the silicon bulk we chose was n-type, which may have resulted in space charge sign inversion (SCSI) under heavy radiation, as in high-energy physics (HEP) experiments [27,28,29]. 

Based on previous work, we simulated the electric characteristics such as electric potential, electric field, and hole concentration. Through analysis and comparison of the distributions on different bias voltages and different positions of thickness, we studied the electric properties more in depth, including the bottom substrate and the full depletion voltage of the detector. Then, the instant induced current before and after radiation was simulated to verify the ultra-fast response speed and the effect of radiation on collection time. As usual, when designing a 3D-trench detector, we placed the p-n junction near the trench electrode to minimize the full depletion voltage. Due to the extremely small size of the device, the detector was fully depleted under a very small bias voltage. In addition, we placed a 20 μm silicon substrate in the bottom of the detector to prevent the detector cell from falling off the wafer during the trench etching process.

## 2. Modeling of the Ultra-Fast 3D-Trench Electrode Silicon Detector on p-Type Bulk 

**Figure 1:**
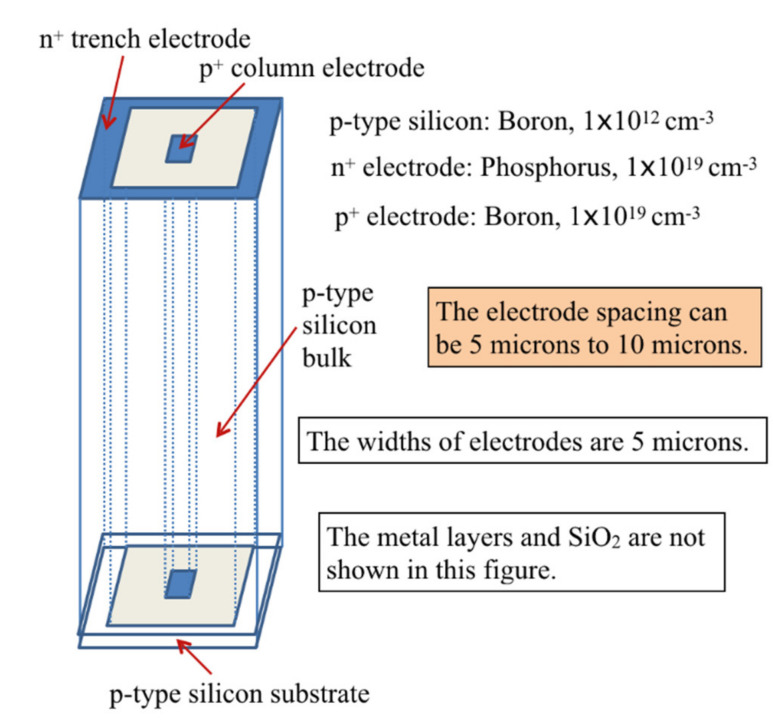
Square ultra-fast 3D-trench electrode detector structure.

For simplicity, we chose square as the shape of our detector cell. Additional practical advantages of the square-shaped cell are that it can easily generate a detector array, and such generated pixel detectors also have a regular shape (square or rectangular) that can be easily diced off the wafer and placed into an application apparatus. The p-type silicon bulk with a thickness in the range of 150–300 µm was doped with 1 × 10^12^ cm^−3^ boron. We chose 200 µm as the detector thickness. The column electrode was p^+^ doped (boron) with a concentration of 1 × 10^19^ cm^−3^, and the 3D-trench electrode was n^+^ doped (phosphorus) with a concentration of 1 × 10^19^ cm^−3^, which placed the p–n junction near the 3D-trench electrode to ensure good electric field distribution and low full depletion voltage. The etching depth (or electrode length) was 180 µm with a width of 5 µm. The electrode spacing was 5–10 µm. There was a 20 µm unetched substrate in the bottom of the silicon bulk to hold the detector effective body (or cell) in the process of etching. On the top of the detector, a 1 µm layer of aluminum (Al) was used to cover the electrodes, and the rest area was covered by a 1 µm silicon dioxide (SiO_2_) layer. The detector bottom was covered entirely by 1 µm of SiO_2_. Figure 1 shows the schematic of a square-shaped ultra-fast 3D-trench electrode detector structure.

## 3. Electrical Characteristics Results

### 3.1. 3D Simulation and Electric Potential Distribution of the Ultra-Fast 3D-Trench Electrode Silicon Detector

**Figure 2:**
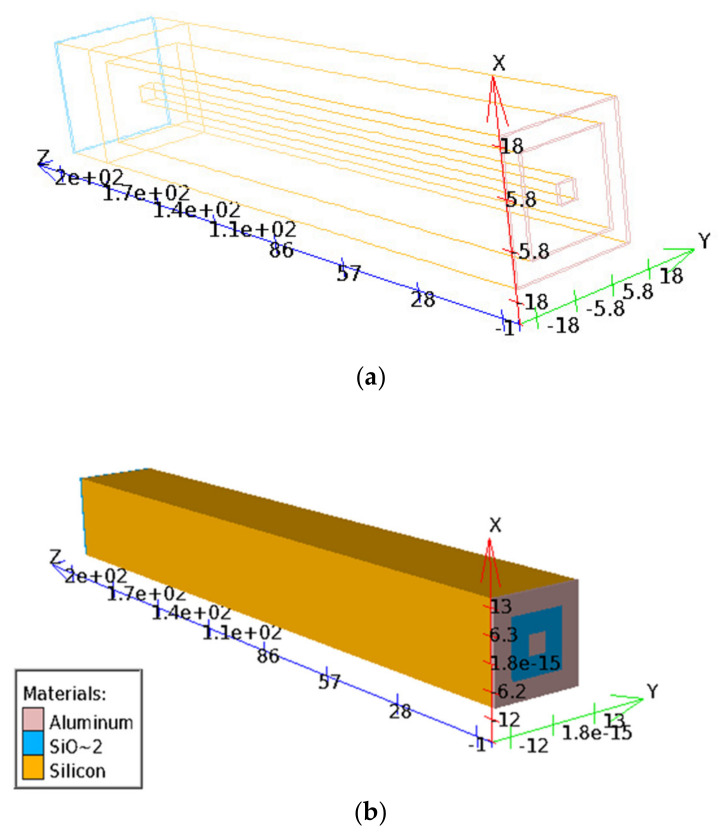
3D simulation of the square ultra-fast 3D-trench electrode detector structure. The axis units are micrometers. The electrode spacings here are (**a**) 10 μm and (**b**) 5 μm.

Simulations in this part were conducted using the Silvaco technology computer-aided design (TCAD) tool [30]. Figure 2 depicts the top view of an ultra-fast 3D-trench electrode detector structure used in our 3D simulation, for which the axis units are micrometers (µm). From Figure 2b, we can see the metal layer and silicon dioxide layers clearly. The whole silicon bulk is presented as well. Cases with electrode spacing of 10 and 5 µm are shown in Figure 2a,b, respectively, with different electrode spacing viewing styles for a better understanding of the detector structure material types. A reverse bias voltage was applied on the column electrode. 

**Figure 3:**
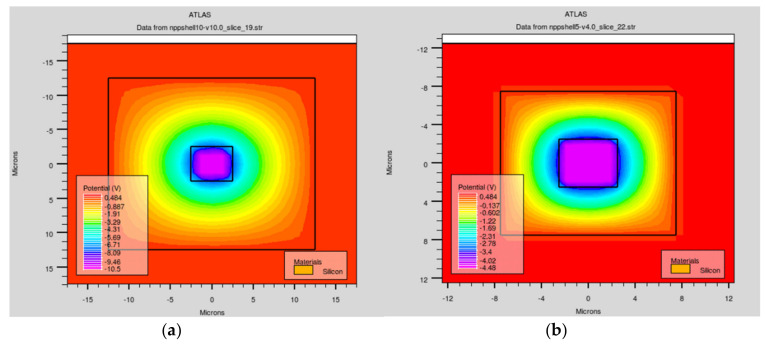
Electric potential profiles along the *z*-axis when z = 103 μm with electrode spacings of (**a**) 10 μm and (**b**) 5 μm.

We constructed a cut plane of the structure at a fixed thickness (z, z = 103 µm) for better displaying the electric potential and field profiles. In Figure 3, the electrode spacings are 10 and 5 µm (Figure 3a,b), the electric potentials are symmetrically distributed in the effective bulk area, and the column electrode is settled at the reverse bias voltage.

**Figure 4:**
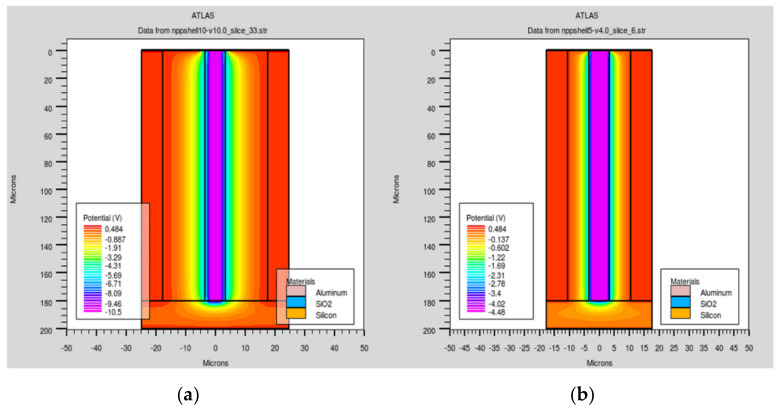
Electric potential profiles along the X–Y diagonal with electrode spacings of (**a**) 10 μm and (**b**) 5 μm.

To clarify the potential distribution near the detector’s bottom substrate, we cut the profiles of the detector on X–Y diagonal planes for detectors with different electrode spacings, as shown in Figure 4. In Figure 4a,b, the electric potentials in the silicon bulk are symmetrically distributed. However, there was potential distribution in the bottom substrate and it was non-uniform. This was especially obvious in the case of the 5 µm electrode spacing, which influenced the electric field distribution and charge collection in this part of detector.

**Figure 5:**
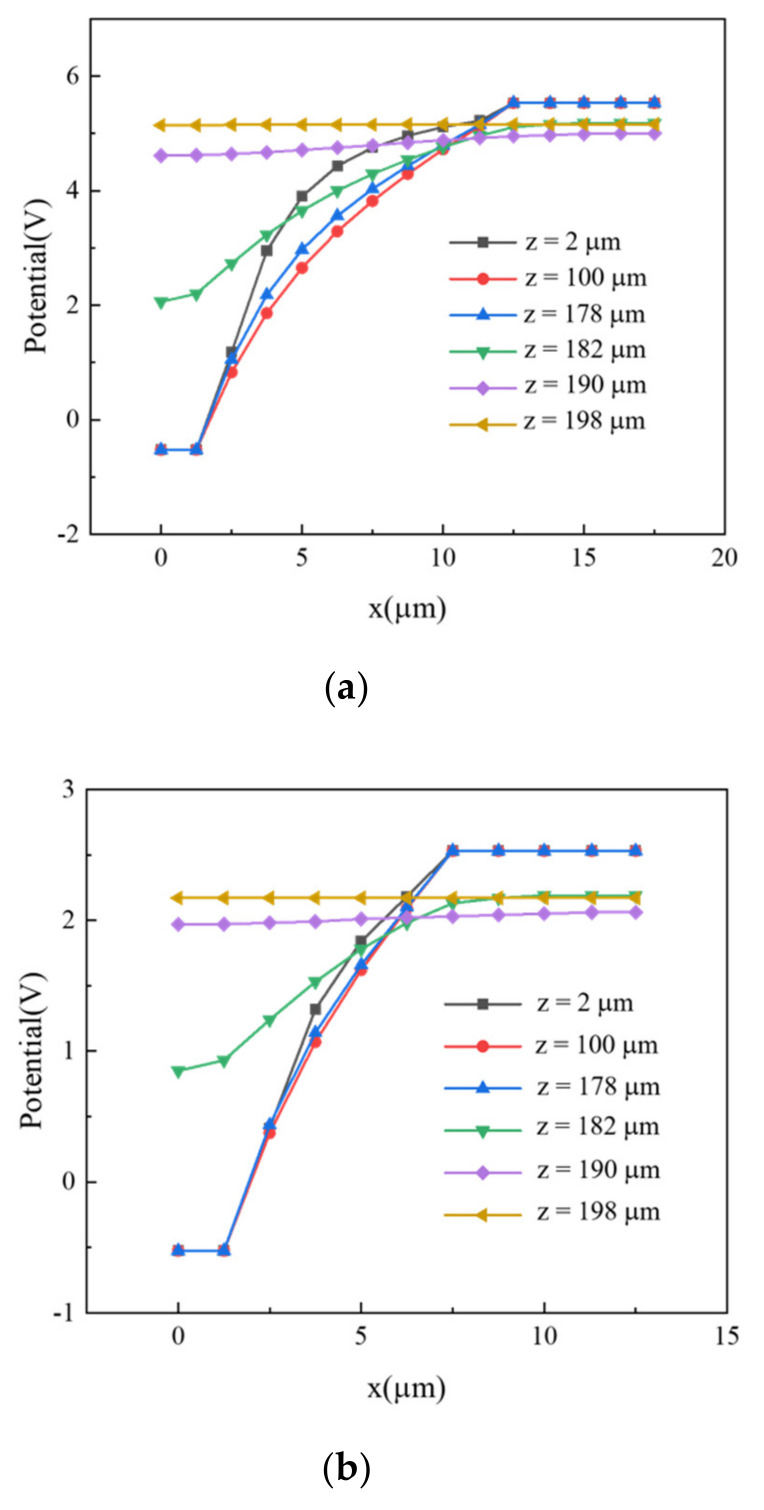
Electric potential curves for different detector structure thicknesses with electrode spacings of (**a**) 10 μm and (**b**) 5 µm.

To further examine the electric potential distributions, we looked at different curves at various z-values (depths along detector thickness) in cut planes shown in Figure 4, with results shown in Figure 5. The curves shown in Figure 5 are electric potential distributions at z-values of 2, 100, 178, 182, 190, and 198 µm. When the cut depth was less than 180 µm, the detector body was surrounded by the trench electrode, and the electric potential value increased along the radius in the detector body. However, when we looked at cut depths over 180 µm thick, the electrodes did not extend into that part of detector, meaning that the electric potential was nearly a constant, indicating low or near zero electric field values near the bottom part of the wafer, the 20 µm unetched substrate.

### 3.2. Electric Field Distribution of the Ultra-Fast 3D-Trench Electrode Silicon Detector

**Figure 6:**
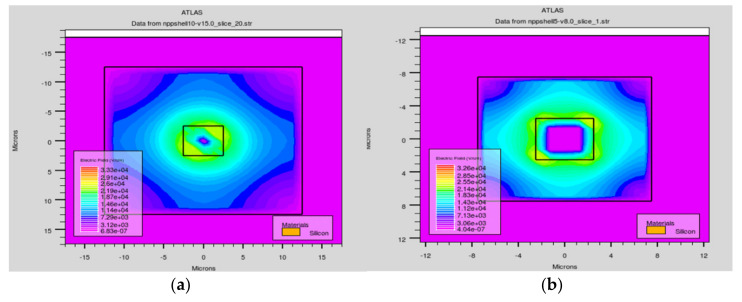
Electric field profiles of the detector along Z axis when z = 103 μm with an electrode spacing of (**a**) 10 μm and (**b**) 5 μm.

**Figure 7:**
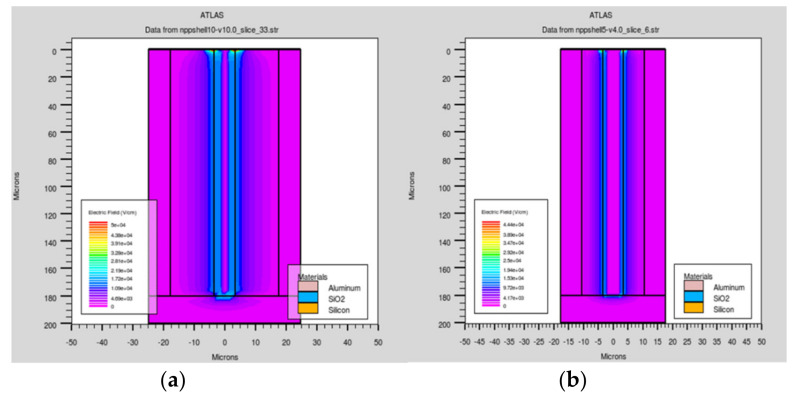
Electric field profiles of the detector along the X-Y diagonal with electrode spacings of (**a**) 10 μm and (**b**) 5 μm.

Another important property of the ultra-fast 3D-trench electrode detector is the electric field distribution. As Figure 6 and Figure 7 show, we made various cut planes to present the electric field profiles of the detector, similar to Figure 3 and Figure 4. Figure 6a,b are the electric field distributions in the cut plane at z = 103 µm for detectors with different electrode spacing. The electric field is larger near the column electrode area than near the trench electrode, and its absolute maximum value is about 2 × 10^4^ V/cm, which is above the value we set to keep the carriers from reaching the saturation drift velocity discussed in our previous work [26]. The electric field distributions are symmetrical.

In Figure 7, the electric field distribution in the X–Y diagonal cut planes for detectors with different electrode spacings is clearly visible. The electric field near the top region of the detector is larger than that of other regions due to this part being directly under the SiO_2_ layer. The electric field is uniformly distributed along the *z*-axis, which ensures a nice charge collection in the entire effective detector body. Holes induced by the incident particle drift horizontally in the electric field towards the central collection electrode. However, the electric field value in the bottom region of the detector (the 20 µm unetched substrate) is lower than that of the bulk, and was seen as a dead area because of the low electric field distribution.

**Figure 8:**
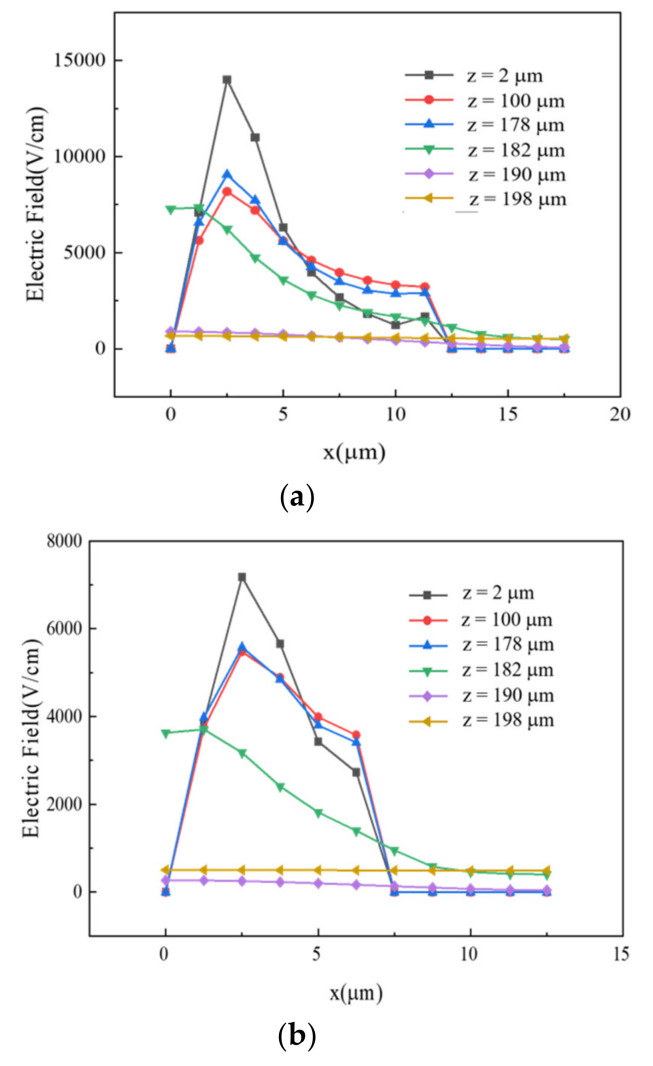
Electric field curves for different detector structure thicknesses with electrode spacings of (**a**) 10 μm and (**b**) 5 μm.

In addition, we studied the electric field profiles along cutlines in Figure 7 at various z-values near the full depletion voltage, shown in Figure 8. Figure 8a shows electric field curves for the detector with an electrode spacing of 10 µm, and Figure 8b shows those for the detector with an electrode spacing of 5 µm. The curves present the electric field at depths of 2, 100, 178, 182, 190, and 198 µm. As mentioned before, there was no extension of the trench electrode for depth below 180 µm. Obviously, electric field profiles were vastly different at different depths. The electric field values near the top SiO_2_ layer (z = 2 µm) were much higher than those in regions far away from the top surface (e.g., for z ≥ 100 µm). Again, this was due to the influence of the SiO_2_ layer, which has a finite positive oxide charge. In the bottom part of the cell, where trench electrode was not extended (z > 180 µm), the values of electric field ere very low. This region was considered a dead region.

### 3.3. Full Depletion Voltage and Leakage Current of the Ultra-Fast 3D-Trench Electrode Silicon Detector

In our previous work on ultra-fast 3D electrode detectors, the main focus was the minimum bias voltage to make the carriers reach the saturation drift velocity [25,26]. The minimum bias voltages we discovered were about 3.5 and 7 V for detectors with electrode spacings of 5 and 10 µm, respectively. The current work, however, focused on the detectors’ full depletion voltages. When the electrode spacing was 10 μm, through simple calculations, the detector’s full depletion voltage was estimated in the order of only a few volts. The exact values of the detector’s full depletion voltage were determined by hole concentration distributions through 3D simulations.

**Figure 9:**
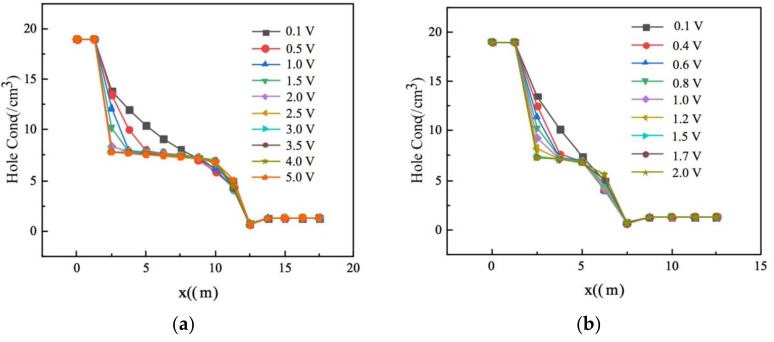
Hole concentration curves (log scale) along *x*-axis for detectors with electrode spacings of (**a**) 10 μm and (**b**) 5 μm.

In Figure 9, we made cutlines along the *x*-axis to produce 1D hole concentration curves. As the bias voltage increased from 0.1 to 5 V, hole concentrations continued to decrease (detector’s depletion regions continue to expand) until full depletion was reached. From this, we surmised that the full depletion value is 1.5 V for the detector with an electrode spacing of 5 µm and 2.5 V for the detector with an electrode spacing of 10 µm.

**Figure 10:**
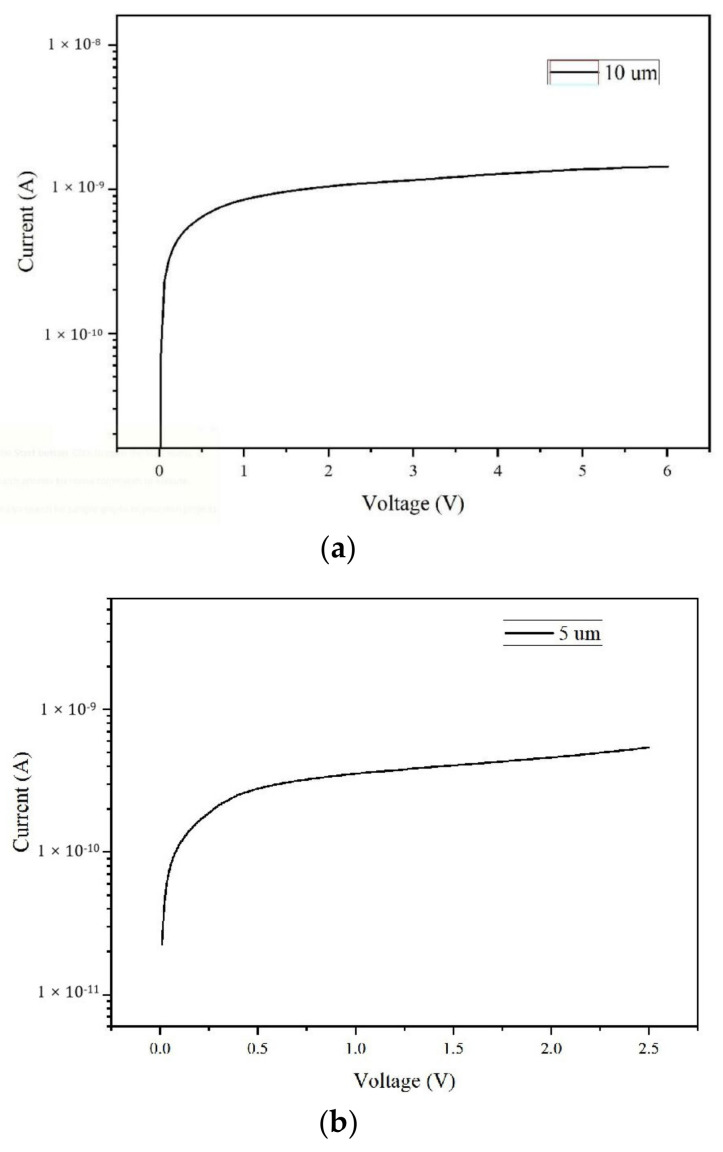
Leakage current curves (log scale) vs. bias voltage for detectors with electrode spacings of (**a**) 10 μm and (**b**) 5 μm.

Figure 10 shows the simulated leakage currents of our 3D-trench detector cell as a function of the bias voltage. Bias voltages and leakage currents are shown with their absolutely values for the purpose of easy reading. From this, we obtained a leakage current at full depletion of 1 × 10^−9^ A for the detector with an electrode spacing of 10 µm and of 3 × 10^−10^ A for the detector with an electrode spacing of 5 µm. Low detector leakage current is essential for certain applications, especially for energy dissipation applications.

### 3.4. Drift Time Study of the Ultra-Fast 3D-Trench Electrode Silicon Detector

In other ultra-fast detector research, the detector thickness averaged about 20–50 μm, which might limit the applicability of the detector for high energy X-ray or particle detection [31,32]. In this work, the detector’s full depletion voltage and other electric characteristics were not limited by the detector thickness due to the special 3D electrode design.

**Figure 11:**
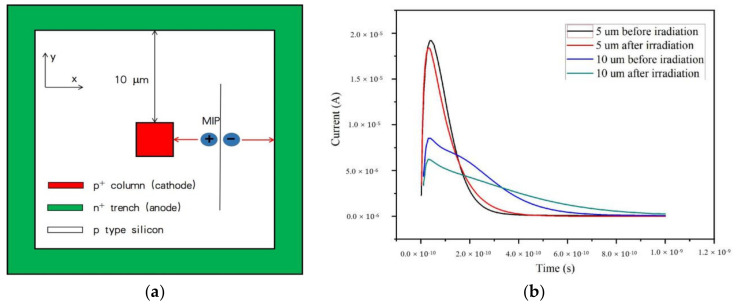
(**a**) Schematic diagram of minimum ionizing particle (MIP) incidence. (**b**) The induced current curves with time before and during radiation with electrode spacing of 10 μm and 5 μm.

We calculated the relationship between the induced current and drift time with a single minimum ionizing particle (MIP) hitting above the detector to demonstrate the ultra-fast property of our 3D-trench electrode silicon detector. We took the structures with spacing of 10 and 5 μm as examples. For the 10 μm spacing, the schematic diagram of MIP incidence is shown as Figure 11a. 

**Figure 12:**
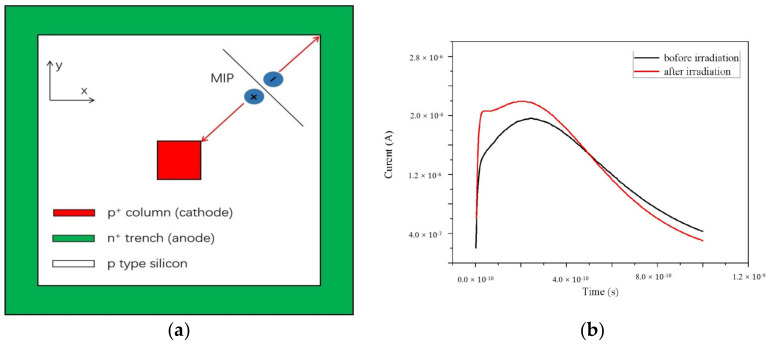
(**a**) Schematic diagram of MIP incidence. (**b**) The induced current curves with time before and during radiation with electrode spacing of 10 μm.

The relationship between carrier drift velocity and electric field intensity and mobility is:

(1) $$v_{dr}^{e,h}=\frac{\mu_{e,h}E}{1+\frac{\mu_{e,h}E}{v_{S}}}$$

where $v_{dr}^{e,h}$ is the drift velocity of electrons/ holes induced by the incident particles and $v_{S}$ is the saturation velocity, taking a value of 1 × 10^7^ cm/s. The carrier drift mobility for electrons, $\mu_{e}$, is 1450 cm^2^/V/s, and the carrier drift mobility for holes, $\mu_{h}$, is 450 cm^2^/V/s. Using the simulation software Sentaurus, the I-t curves were simulated and presented in Figure 11b and Figure 12b, with different induced positions shown in Figure 11a and Figure 12a. The radiation fluence was set to 1 × 10^16^ n_eq_/cm^2^.

The induced current signal on detector electrodes was based on the Shockley–Ramo theorem

(2) $$i=q\cdot {\vec{E}}_{w}\cdot {\vec{v}}_{dr}=q\cdot \left(\left|{\vec{E}}_{wx}\right|{\vec{v}}_{x}+\left|{\vec{E}}_{wy}\right|{\vec{v}}_{y}+\left|{\vec{E}}_{wz}\right|{\vec{v}}_{z}\right)$$

where i is the induced current, q is the charge of the carrier, ${\vec{E}}_{w}$ is the weighting field, and ${\vec{v}}_{dr}$ is the carrier drift velocity, which depends on the internal electric field of the detector.

In Figure 11b, the peak point is 3.68 × 10^−11^, 1.92 × 10^−5^ before radiation, and 3.15 × 10^−11^, 1.84 × 10^−5^ after radiation when the electrode spacing is 5 μm. When the electrode spacing is 10 μm, the peak point is 3.00 × 10^−11^, 8.52 × 10^−6^ before radiation, and 3.00 × 10^−11^, 6.24 × 10^−5^ during radiation. From the calculation above and simulation results, the maximum drift time of electrons and holes are in the order of tens to hundreds of picoseconds for a detector with an electrode spacing of 10 μm at bias of −10 volts. As we increased the bias voltage further to $\left|V\right|$ > 10 volts, carrier drift time decreased further. Figure 11b shows that radiation may influence the drift time slightly. The drift time may decrease after radiation due to the radiation-induced decrease of effective carriers’ drift distance.

We simulated another induced current curve with a single MIP hitting on the diagonal position of the square, pictured in Figure 12a, and Figure 12b shows the results. In Figure 11, the curves are similar for electrode spacing of 5 and 10 μm; therefore, we simulated the 10 μm structure only in this part. In Figure 12b, the peak point is 2.40 × 10^−10^, 1.96 × 10^−6^ before radiation and 1.40 × 10^−10^, 2.15 × 10^−6^ during radiation. The peak induced current value decreased likely because the field on the diagonal was lower.

## 4. Detector Array of the Ultra-Fast 3D-Trench Electrode Detector Cells

**Figure 13:**
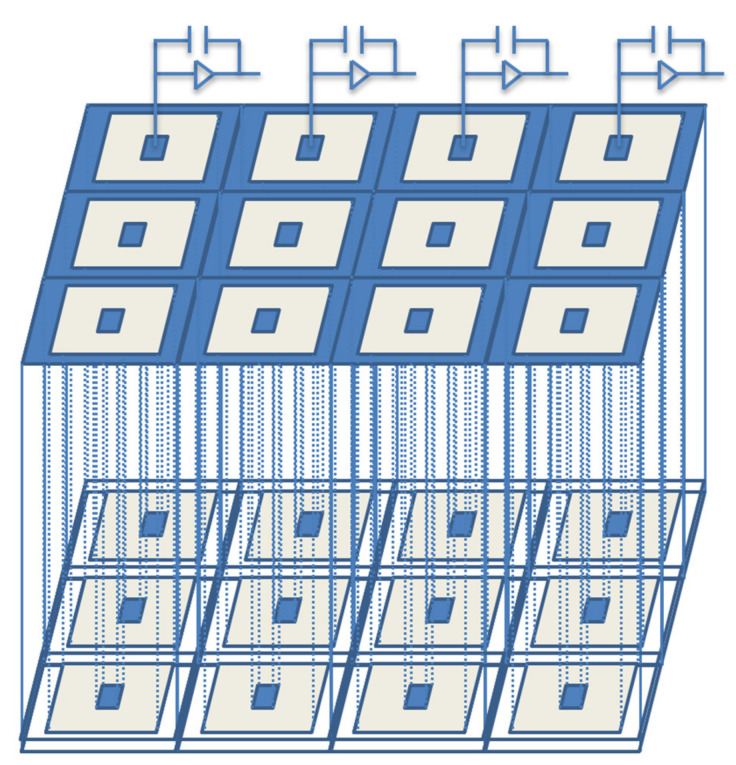
The schematic of a 4 × 3 array of the ultra-fast 3D-trench electrode detector cells.

The electrode spacing of the ultra-fast 3D-trench electrode silicon detector on p-type bulk is only 5–10 µm. When these detector cells form a detector array, it can be used as a high-resolution, position-sensitive detector. These detector arrays, or pixel detectors, can be applied in medical imaging, high-energy physics experiments, and other high-radiation environments due to their high position resolution, high radiation hardness, and ultra-fast response time. Figure 13 shows a schematic of a 4 × 3 detector array. Each pixel is connected to a readout channel that is connected to an application specific integrated circuit (ASIC). In order to keep the picture viewable, we did not draw all of them. Adjacent detector cells can share the same trench electrode, which reduces the dead region considerably, thus improving the detector performance significantly. 

## 5. Conclusions

In our previous study on ultra-fast 3D-trench electrode detectors, we investigated the cross-section of electric potential and field distribution to estimate the minimum bias voltage to make carrier drift velocity reach saturation velocity, established the structure overcome the central low electric field region (dead area) of a 3D-column electrode detector, and then studied the breakdown situation. However, to simplify the analysis, we did not have a substrate in the bottom of the device structure.

In this work, for the purpose of simulation under radiation, p-type silicon detector bulk was chosen to avoid SCSI in heavy radiation environments. We verified that only under a few volts, in most of the detector effective area (silicon body), electric field values are enough for free carriers to reach saturation drift velocity, and we investigated the electric potential and field distribution in the bottom substrate part, which will provide support for our future fabrication and measurements. We measured very low leakage currents and full depletion voltages from simulation results, which promotes many applications of the detector. Lastly, the induced current curves with drift time were simulated, from which we verified that the response/drift times of the detector are in the order of tens to hundreds of picoseconds with electrode spacing of 5 or 10 μm.

The detector cells can form a detector array to be used as a position-sensitive pixel detector with extremely high resolution due to the small size and ultra-fast response time of the detector cells. Detector arrays will be studied in further detail in the future.
